# Childhood Trauma, the HPA Axis and Psychiatric Illnesses: A Targeted Literature Synthesis

**DOI:** 10.3389/fpsyt.2022.748372

**Published:** 2022-05-06

**Authors:** Felim Murphy, Anurag Nasa, Dearbhla Cullinane, Kesidha Raajakesary, Areej Gazzaz, Vitallia Sooknarine, Madeline Haines, Elena Roman, Linda Kelly, Aisling O'Neill, Mary Cannon, Darren William Roddy

**Affiliations:** ^1^Department of Psychiatry, Royal College of Surgeons in Ireland, Dublin, Ireland; ^2^Department of Psychiatry, Trinity College Institute for Neuroscience, Trinity College Dublin, Dublin, Ireland; ^3^Department of Anatomy, Trinity College Dublin, Dublin, Ireland

**Keywords:** childhood adversities, HPA axis (hypothalamus–pituitary–adrenal), depression, psychosis, development

## Abstract

Studies of early life stress (ELS) demonstrate the long-lasting effects of acute and chronic stress on developmental trajectories. Such experiences can become biologically consolidated, creating individual vulnerability to psychological and psychiatric issues later in life. The hippocampus, amygdala, and the medial prefrontal cortex are all important limbic structures involved in the processes that undermine mental health. Hyperarousal of the sympathetic nervous system with sustained allostatic load along the Hypothalamic Pituitary Adrenal (HPA) axis and its connections has been theorized as the basis for adult psychopathology following early childhood trauma. In this review we synthesize current understandings and hypotheses concerning the neurobiological link between childhood trauma, the HPA axis, and adult psychiatric illness. We examine the mechanisms at play in the brain of the developing child and discuss how adverse environmental stimuli may become biologically incorporated into the structure and function of the adult brain *via* a discussion of the neurosequential model of development, sensitive periods and plasticity. The HPA connections and brain areas implicated in ELS and psychopathology are also explored. In a targeted review of HPA activation in mood and psychotic disorders, cortisol is generally elevated across mood and psychotic disorders. However, in bipolar disorder and psychosis patients with previous early life stress, blunted cortisol responses are found to awakening, psychological stressors and physiological manipulation compared to patients without previous early life stress. These attenuated responses occur in bipolar and psychosis patients on a background of increased cortisol turnover. Although cortisol measures are generally raised in depression, the evidence for a different HPA activation profile in those with early life stress is inconclusive. Further research is needed to explore the stress responses commonalities between bipolar disorder and psychosis in those patients with early life stress.

## Introduction

Alterations of the hypothalamic–pituitary–adrenal (HPA) axis is one of the most notable neurobiological findings in psychiatry (1, 2). However, HPA changes are inconsistent both across and between psychiatric conditions. It is thought that early life stress (ELS) may influence subsequent HPA development and subsequent responses to stress, resulting in a vulnerability to psychiatric illness. Development of the human brain is complex, and is characterized by dynamic stages of plasticity and periods of complex biological and environmental integration. However, while early experiences have the capacity to shape the brain and give rise to vital developmental competencies such as language there is an equal potential for adverse exposures such as trauma-related stress to cause maladaptive developmental changes. Experiences of adverse events can become biologically consolidated, creating individual vulnerability to an array of psychological issues later in life (3, 4).

In this review, we explore the developmental trajectory of the child and the psychopathological consequences of early childhood trauma. We also discuss the normal HPA-axis stress response and a targeted review of HPA-related findings in mood disorders and psychosis.

## Early Life Stress and Trauma

### Defining and Measuring Early Life Stress and Trauma

Stress may be classified into three distinct categories (5). “Positive stress,” referring to normative and short-lived adverse experiences with minor physiological change: e.g., meeting new people or changing daycare. “Tolerable stress,” as characterized by intense but short-lived adverse experiences such as the death of a family member or a natural disaster. The effects of this stress are thought to be reversible when accompanied by substantial support from a parent/adult, however tolerable stress may progress into the final subtype; “toxic stress.” Toxic stress is defined as intense adverse experiences enduring over a longer period, spanning weeks, months or years such as war, natural disasters, and physical, emotional and sexual violence. This form of stress may result in permanent physiological and psychological changes to the developing child. Although the terms “stress” and “trauma” have been used interchangeably in the literature, trauma can be more specifically associated with “toxic stress.”

Various tools designed to assess adverse childhood experiences of stress and trauma exist (6). Using the Adverse Childhood Experiences (ACE) study, Felliti et al. designed a questionnaire examining the effects of psychological, physical and sexual abuse, alcohol and drug exposure, health related problems, and depression. They found that instances of toxic stress can be divided into three subtypes: abuse (physical, sexual, emotional), neglect (physical, emotional), and household dysfunction (domestic violence toward a parent, household substance abuse or other mental illness, problematic separation/divorce). These toxic stress/traumatic events in turn demonstrate strong associations with multidimensional negative health outcomes in adulthood, including adverse mental health issues, ischemic heart disease, cancer, chronic lung disease, skeletal fractures, and liver disease. Since the development of the ACE questionnaire, tools with greater specificity and validity have been developed, such as the Childhood Trauma Questionnaire (7) and The 27-item Early Trauma Inventory Self-Report-Short Form (ETISR-SF) (8). Studies consistently demonstrate associations between poor health outcomes and childhood stress and trauma in the absence of protective factors (9).

### Prevalence of Childhood Trauma

Epidemiological studies have shown a moderate to high prevalence of childhood trauma across populations. In the United Kingdom, 16% of children report having experienced trauma (10), but figures climb as high as 32% in Canada (11). However, a recent systematic review, recommends caution in interpreting national prevalence rates, detailing that emotional abuse in childhood is as common as 83% in Greece (12). These prevalence rates are a major cause of concern given the strong evidence supporting the persistent harmful effects of early traumatic experiences on adult psychological wellbeing (13), and the significant association between childhood trauma and the later occurrence of psychiatric disorders such as major depressive disorder (MDD), bipolar affective disorder (BD) and psychosis (2, 14, 15). Although there is a large body of research demonstrating that early childhood trauma is an important risk factors for psychopathology (16), a concrete understanding of the psychobiological processes behind this link remains limited. One potential mechanism is the disruption in the development of the stress response system during childhood, and its dysregulation into adulthood.

### The Neurosequential Model of Neurodevelopment

According to the neurosequential model (17), the development of complex interrelated brain structures occurs sequentially and hierarchically. That is, the more complex and dispensable a system is to immediate survival, the later it develops. Regions involved in cardiorespiratory actions are fully functional at birth (e.g., the brainstem), while regions involved in higher executive functions such as emotional and behavioral regulation (e.g., the prefrontal cortex) require longer periods to organize and develop fully. The micro neurodevelopmental processes (i.e., synaptogenesis, myelination, migration, differentiation, arborization, and apoptosis) are scheduled within the developmental trajectory with a prearranged plan, with different brain areas developing at time-specific periods. However, these developmental processes are not independent of each other, and as such, factors influencing early neural development are likely to result in dysfunctions that also affect later development of higher cortical and limbic areas (18).

Implications of the neurosequential model indicate that throughout human development, there are periods when certain biological systems are more malleable in response to environmental stimuli. These sensitive periods can be defined as time frames (or windows) during which developmental systems are vulnerable to certain stimuli (17, 19).

### The Trajectory of a Sensitive Period

To demonstrate the importance of sensitive periods it is important to first understand how they operate. A sensitive period is triggered by intense neural activity initiated by an experience (20). This phenomenon can be characterized as the “opening” of a sensitive period, a point from which the developing system is receptive to environmental stimuli. The termination or “closing” of a sensitive period is less well understood. A review by Johnson (21) describes the termination phase of a sensitive period as a period of significant decline in plasticity (21), suggesting potential explanations: (1) termination arises from endogenous factors controlled by biological maturation or external environmental triggers; (2) learning is self-terminating; and (3) underlying plasticity does not reduce, but rather the constraints on plasticity become stable.

According to the first view, maturational and/or environmental factors cause neurochemical changes in certain brain areas which increase the rate of pruning leaving strong existing patterns in full functional capacity. Areas with strong synaptic connectivity become permanently linked, and therefore indicate the end of a sensitive period. Several sensitive periods appear to end as an animal (or human) approaches sexual maturity (20). For instance, heightened plasticity in the sound localization pathway in barn owls declines as these juveniles approach adulthood (22).

The second view proposes that the process of learning may produce changes in the brain that reduce the system's overall plasticity (23). Studies using computer-simulated neural networks (24) support the view that unspecialized brain systems have higher levels of plasticity, meaning that the connections within these systems are sensitive and adaptable. As the system specializes and changes, it becomes rigid and less sensitive as a result. For instance, it is more difficult to learn a second language as an adult when a primary language has already been learnt. This means that learning in one particular way impedes learning in another and, therefore, reduces plasticity. Unless earlier-learned abilities are neglected or lost, new learning may always be limited.

The third view suggests that plasticity does not reduce but rather the constraints of plasticity become stable. For example, Thomas and Johnson (23) describe the change in information received by visual cortex regions as the distance between an infant's eyes increases (23). To keep up with the increasing distance between the eyes, the cortical areas of the brain must remain malleable. However, plasticity does not decline once growth stops. Instead, it becomes constrained by fixation of the eyes once the child stops growing. Thus, the plasticity becomes “hidden” by the features that constrain it.

A further conceptual model known as the “stress acceleration model” argues that experiences of toxic stress or trauma may lead to faster (or accelerated) maturation of the neural circuits responsible for emotional processing and is therefore evidence of early system adaptation (25). The model suggests that support from a caregiver enables the child to develop emotional circuits following a normal developmental pattern. In the absence of this support, development of emotional circuitry is forced to accelerate. While this is potentially adaptive in the short term, premature closure of the sensitive period for emotional development may lead to poor emotional functioning in the long term (26).

#### Reopening Sensitive Periods

Recent work has also explored the possibility of “reopening” sensitive periods. For example, MDMA has been found to reopen a striatal-sensitive period involved in social reward learning in rodents (27). Fluoxetine (a treatment for MDD) can increase plasticity of the visual cortex in patients with amblyopia (28). Antidepressants are also associated with increased neurogenesis in the hippocampus (29) and may a potential mechanism for antidepressant effect. However, suppression of plasticity by fluoxetine elsewhere has also been reported (30). Valproate (a treatment for epilepsy and BD) can reopen auditory sensitive periods for determining absolute pitch (31). The reopening of sensitive periods for emotional circuitry such as those compromised in the HPA-axis are yet to be investigated.

### The Stress Response

The human response to a threat results in hyperarousal; facilitated by the sympathetic nervous system. This hyperarousal can be viewed on a continuum, including states of calmness to arousal to alarm, fear, and terror – this final stage commonly referred to as the “fight, flight or freeze” response (32). Hyperarousal causes physiological changes such as increases in blood pressure, heart rate and respiration, cognitive changes such as hypervigilance and detachment from unessential environmental cues, and an initiation of outward behaviors such as crying or shouting (33). While these responses are an adaptive mechanism in adults, it is not as useful for children or infants who lack the physical capabilities to flee or defend themselves. Instead, the primary purpose for this response in children is to attract a primary caregiver who can protect or remove them from the situation. Importantly, the traumatic stress response (i.e. the “toxic stress response” response) differs from the regular stress response in that the neurochemical changes, which are initially beneficial, often outlive the threat of the stressor. This means that the hyperarousal state continues even when the stressor has dissipated. This becomes problematic, causing disruptions to homeostasis, and the emergence of a maladaptive feedback cycle (34). A complex set of neurobiological interactions underlie this process. While researchers differ between which brain areas are included in the model of the traumatic stress response in children (4, 35–38), most agree that it is embedded within three major circuits: the HPA axis, the limbic system, and the prefrontal cortex.

### The HPA Axis

The HPA axis represents the major neuroendocrine stress response system that serves to adapt an organism to demanding change. The release of Corticotropin Releasing Hormone (CRH) by the hypothalamus induces alertness and increased attentional capacity. CRH prompts the release of adrenocorticotropic hormone (ACTH) from the pituitary, which subsequently induces the secretion of adrenal cortisol and cortisone. Under normal conditions, cortisol is released with a distinct diurnal rhythm, characterized by levels increasing during the night just prior to waking, a transient acute spike in release following awakening (the cortisol awakening response), followed by a steady decline throughout the day until sleep. Physical or psychological stressor also induces a temporary spike of cortisol (39). In contrast, reduced morning cortisol levels have repeatedly been observed in chronically stressed individuals (40–42). This may be due to a protective downregulation of the HPA axis to avoid overexposure to stress hormones (43).

A potential mechanism underlying the mediating effects of adverse experiences during childhood on adult psychopathology has been seminally modeled in McEwen's (44) theory of Allostatic Load (44). This theory posits that chronic stressors cause a long-lasting deviation of the normal stress state, resulting in a new established set point. This predisposes an individual to increased vulnerability for developing pathologies, both physical and psychological (45).

Pituitary volume changes have been reported in MDD with both increased (46, 47), reduced or not change in volume reported (48–50). In contrast, BD is mostly associated with reduced pituitary volumes (51, 52). Similarly, larger pituitary volumes were found in first episode psychosis (53, 54), clinical high risk individuals and those with a family history of psychosis (55). Larger volumes in the at risk groups were found in those who later transition to psychosis (53, 56). In contrast, smaller volumes are found with chronic schizophrenia (57, 58), possibly reflecting pituitary hypoplasia following repeated HPA overactivity. ELS has been found to be a predictor of increased anterior, but not posterior pituitary volume in adolescents.

**Figure 1:**
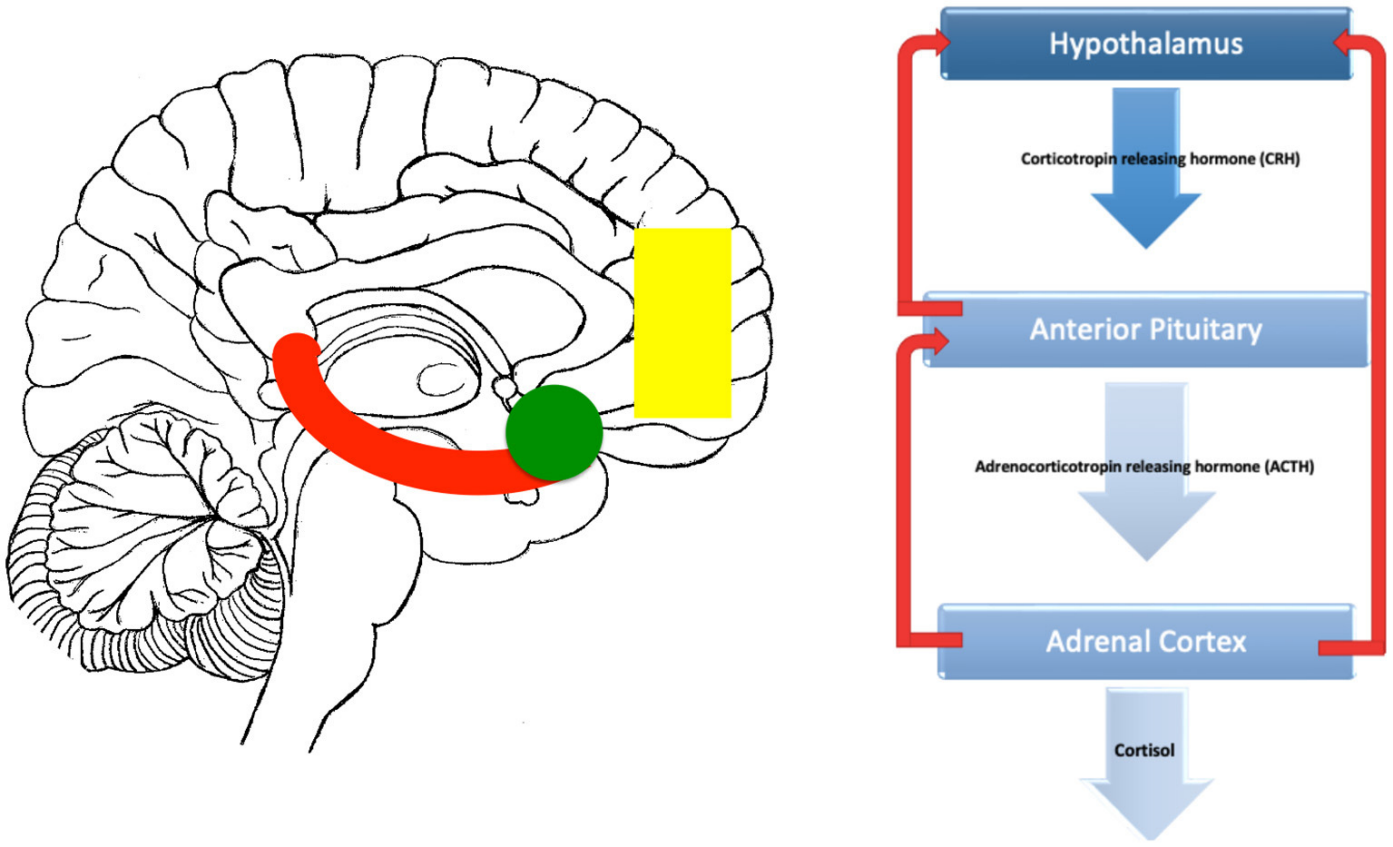
Brain related areas and the HPA axis. On the left is a midline saggital representation of the brain showing a stylised hippocampus (red), amygdala (green) and medial prefrontal cortex (yellow). On the right, the classic HPA axis is shown. Inputs to the HPA from the hippocampus are mostly inhibitory, inputs from the amygdala are mostly excitatory while the prefrontal inputs can be both inhibitory and excitatory depending on whether they originate from the dorsal (inhibitory) or vental (excitatory) prefrontal areas.

Although the HPA axis is regulated internally through negative feedback loops withing the axis itself, it is also recieves dense connections from brain regions involved in the processing of stress. This regions in include the limbic system and medial prefrontal cortex (mPFC) (Figure 1).

### Limbic Control of the HPA Axis

The Limbic System is a group of interconnected brain regions involved in emotion, memory.and behavior (59). The amygdala and hippocampus are key hubs within the limbic system (60). The amygdala assigns emotional valence to sensory inputs (61), whereas the hippocampus has roles in memory formation (62). Both structures have connections with the HPA axis, allowing modulation of the stress response. While the amygdala is principally excitatory to HPA axis functioning, the hippocampus is mostly inhibitory (63–65).

#### Amygdala

The amygdala lies anterior to the hippocampus in the medial temporal lobe and consists of three functional nuclear divisions: the central, basolateral, and corticomedial groups. The HPA axis is largely influenced by the central and corticomedial groups which project in abundance to the hypothalamus. Lesions to these groups have been shown to reduce ACTH and corticosterone secretion following stress (66, 67). Stimulation to central nuclei causes overexpression of CRH resulting in an increase in HPA axis activity (68–70). Amygdalar effects of the HPA axis have also been shown to be region and stressor specific (71, 72).

Induced early life stress has been associated with amygdalar hypertrophy in primates (73), with increased volumes also found in children exposed to both neglect (74) and mild maternal disengagement (75). Interestingly, no change in amygdala volume has been found in individuals exposed to physical or sexual abuse as children (76, 77). Reviews have found that amygdalar volumes are reduced bilaterally in MDD (60) and in pediatric BD (78) with varying amygdalar connectivity with prefrontal regions in both conditions (79). Reduced amygdalar volume has been suggested as a mechanism for stress sensitization to MDD following exposure to violence in children (26). The evidence for amygdalar volume differences in psychosis is more nuanced (80) with some studies of first episode psychosis revealing complex volume reduction (81–83) while others did not show significant differences (84). Similar results were found in patients with schizophrenia (79, 85, 86). The experience of childhood trauma has been found to be predictor of right and total amygdalar volumes in first episode psychosis (87) (Hoy). Perturbations in amygdalar inputs to the HPA axis in the developing brain due to ELS may presdispose to MDD, BD and psychosis.

#### Hippocampus

The hippocampus inhibits the HPA axis through its fornix outputs to the hypothalamus (59, 63, 64). Hippocampal stimulation decreases glucocorticoid secretion in rodents (88) and multiple studies suggest that rodents genetically modified to have reduced hippocampal function results in dramatic increases in corticosterone release (89, 90). However, regulation of the HPA axis by the hippocampus appears to be both region- and stressor-specific. Trauma also results in significant hippocampal changes (91) with ELS decreasing adult hippocampal neurogenesis in rodents (92). Smaller hippocampal volumes have been found in individuals with Post-traumatic Stress Disorder (PTSD) (93), and pre-clinical models showing significant reductions in rodent hippocampal volume following induced stress compared to pre-stress size (94). Both depression and stress in people with chronic pain have also been shown to modulate hippocampal metabolite function (95–97). Despite pre-clinical studies suggesting acute changes resulting from stress, it has been suggested that reduced hippocampal volume is not the result of, but rather a risk factor for conditions such as PTSD (98).

Reduced hippocampal volumes is the most reported finding in MDD (99–103) including in depressed children (101), indicating that hippocampal volume changes may be an early marker for MDD. Childhood trauma has been associated with smaller hippocampal regions in MDD comparted to those without childhood trauma (36). Most brain imaging studies have found no changes in hippocampal volume in BD (104–106), but some studies have reported reduced volumes, (107–110). Interestingly, Childhood trauma is associated with increased amygdala gray matter volume patients with BD compared to those without trauma (111).

In contrast, reduced hippocampal volume is an established finding in schizophrenia (82, 108, 112–117), Individuals at ultra-high risk for the development of psychosis may also demonstrate reduced hippocampal volumes (80, 83). Childhood trauma has been found to be predictor of left hippocampal volume in first episode psychosis.

### Prefrontal Cortex Control of the HPA Axis

The medial prefrontal cortex (mPFC) regulates the response of the amygdala by processing additional sensory information experienced during a traumatic event (38). Structural changes have also been observed in the mPFC in patients with PTSD (118, 119). The prefrontal cortex has an extended sensitive period and continues to develop into early adulthood, making it more susceptible to insults through childhood to adolsecence. Dysregulation in developing executive functional capacities during childhood may impact the processing of both traumatic and non-traumatic situations in the future. Reduced mPFC volume has repeatedly been demonstrated in adults reporting childhood emotional maltreatment and/or early life adversity (120, 121). This has significance for a range of psychological and psychiatric conditions given the vital role of the medial prefrontal cortex in the “top down” regulation of emotional behavior. Lesions of the cingulate gyrus are linked to enhanced ACTH and corticosterone secretion in rats (122). Other studies imply that the role of the mPFC is substantially more complex. Lesions of the right infralimbic cortex decrease corticosterone responses to restraint stress, while lesions restricted to the left do not affect glucocorticoid secretion at all (123). Additionally, induced ELS via the maternal separation model reduces pre- and post-synaptic protein expression of inhibitory neurons in the mPFC (124). Importantly, however, is that the cingulate gyrus and infralimbic cortex efferently project to different brain areas. The cingulate cortex projects to stress inhibitory (dorsomedial hypothalamus and the paraventricular hypothalamic nucleus), whilst the infralimbic cortex projects to stress excitatory areas (stria medullaris and amygdala) (125–128). Overall, these observations suggest that different mPFC areas are associated with different roles in HPA axis regulation (129).

Children diagnosed with preschool onset MDD have reduced right ventromedial PFC volume compared to controls (130). Many studies have have identified abnormal amygdala-PFC functional connectivity in MDD compared to controls (131–134). Similarly in BD, amygdala-PFC functional connectivity abnormalities have been reported (135). Like MDD, there are also reports of amygdala-PFC connectivity abnormalities in BD (136) and psychosis (137).

### Childhood Trauma Causes Adult Sensitization of the HPA Axis

The process of HPA axis sensitization to stress as a result of childhood trauma may occur long before adulthood. Dysregulated cortisol responses are detectable in adolescents with a history of child abuse (138) and exposure to childhood violence (139). Importantly however, dysregulated responses are not consistently associated with psychopathology such as MDD or PTSD. This suggests that the onset of psychopathological symptoms, due to neurobiological changes, may be occurring later in the developmental trajectory.

A wide disparity exists across studies investigating HPA axis reactivity in adolescents who have experienced childhood trauma. While some studies report hyperactivation of the HPA axis in response to stress (3) others report hypoactivation (138, 140). Likewise, inconsistencies are observed for diurnal regulation (141, 142). A potential explanation for these contradictory findings has been provided by Kuhlman et al. (143). In their study, adolescents completed the Socially Evaluated Cold Pressor Task (144) while their parents completed the Early Trauma Inventory (8). Salivary cortisol samples were taken from the participants as part of the stress test procedure in addition to 1 week later during 2 consecutive weekdays as a measure of diurnal rhythm. Results indicated that exposure to “non-intentional trauma” (e.g., witnessing an accident or experiencing a natural disaster) was associated with normal diurnal regulation but elevated cortisol at bedtime. “Physical abuse” (e.g., being injured to the point of bruising) was associated with faster reactivity to acute stress. “Emotional abuse” (e.g., persistently being ridiculed or insulted by a caregiver) was associated with delayed recovery following acute stress. The authors suggest that HPA axis functioning can be perceived as specific to trauma subtypes rather than inconsistent across studies; however, more research to establish a concrete connection between reactivity and subtype in adolescents.

An important question remains, will these findings generalize to adult sensitization and will such sensitization result in psychopathology? A retrospective review of current rodent models shows promising results in favor of this relationship. Recreations of early life neglect (maternal separation and/or early weaning) in rodent offspring have allowed for insight into the potential long-term biological and behavioral effects of trauma. Neglected rodent offspring have shown increased susceptibility to anxiety and depressive-like behaviors when exposed to stress in both adolescence and adulthood (145, 146). Similarly depressive-like behavior in neglected female rodents was found when faced with the forced swim test (147). The same rodents also exhibited significantly elevated corticosterone levels, indicating a dysregulated HPA axis response and, thus, heightened sensitivity to stress. Moreover, the preliminary evidence observed throughout these various rodent models suggests that early life stress does indeed cause adult sensitization to stress and likely causes a predisposition to psychopathologies such as mood disorders and psychosis.

## The HPA Axis, Early Life Stress and Psychiatric Conditions

Early life stress (ELS) due to childhood abuse and/or neglect has been linked to increased risk of psychiatric illness onset and recurrence, increased disease severity and poor treatment response (pharmacotherapy and psychotherapy) (148, 149). The remainder of this review will target key articles regarding HPA activity and early life stress in mood, anxiety and psychotic disorders. Studies investigating daily cortisol secretion (eg, morning, total daily cortisol etc.) and the cortisol response to both awakening and stressors will be examined in these disorders, with an emphasis on recent metaanalyses where appropriate. The HPA activation directly after awakening is known as the cortisol awakening response (CAR) and involves a transient “bump” in cortisol between 30 mins to an hour after awakening, usually measured as area under the curve (AUC) from sequential testing during the first hour after awakening. The CAR is thought to provide a measure of the reactivity and reserve of the HPA axis. The cortisol response to stressors involve before and after measurements and are often also described as AUC. Common stressors used in these studies include the Trier Social Stress Test (TSST) and other psychological stressors. HPA responses following physiological manipulation, e.g., dexamethasone suppression (DST) will also be examined.

### Mood Disorders

MDD and BD are the most common mood disorders and are both associated with poor life quality, increased disability, and mortality (150). In the United States, MDD and BD have a lifetime prevalence of 16 and 5% respectively (150). An MDD episode presents with depressed mood and/or anhedonia (diminished interest or pleasure) with a collage of other symptoms including psychomotor and sleep changes. BD has two distinct pathological phases, a depressed phase similar to an MDD episode and an mania or hypomanic phase presenting with periods of elated mood and increased energy. A systematic review of 44 articles looking at different subtypes of ELS (sexual abuse, physical abuse, emotional abuse, physical neglect, and emotional neglect) concluded that mood disorders are associated with all forms except emotional neglect (151).

#### High Daily Cortisol Is Not Specifically Associated With MDD and ELS

A case-control study found that childhood trauma severity was not associated with high diurnal salivary cortisol (based on AUC, measured at awakening, noon and 8 p.m.) in currently depressed MDD patients with although this association was present in patients with glucocorticoid resistance (152). Another study of early adolescent females, aged 9 to 14, with a genetic predisposition to depressive illness, had higher daily cortisol if they had experienced maltreatment during their childhood (153). Although no differences were demonstrated in long term hair cortisol assays between those with childhood trauma vs. those without, patients who were unresponsive to treatment revealed lower cortisol levels prior to psychological treatment (154). A further study investigating differences in baseline cortisol in patients with comorbid psychiatric illnesses found no baseline cortisol differences in either children or adults with comorbid MDD and PTSD (155).

A meta-analysis of 651 depressed children and adolescents found greater basal cortisol levels in MDD children and adolescents compared to controls (156). Higher morning cortisol was reported in a meta-analysis of 1,354 depressed adult patients. An attenuated effect was observed for the evening salivary cortisol, however this was based on a smaller number of studies, all of which were underpowered (157). A further metaanalysis of 18,374 adult individuals found higher cortisol in MDD when measured continuously throughout the day, with morning times revealing the least difference between the groups. Morning MDD CRH was also found to be higher compared to controls. Interestingly, removing the one study (of 16 CRH studies) with the largest effect size collapsed this CRH increase. ACTH was also higher overall in MDD patients; however, there was no difference between control and depressed patients at any one time of day (158). Similarly, another meta-analysis of 727 patients over 60 years old observed higher basal morning cortisol in patients. Morning ACTH also showed no difference between groups (159). A meta-analysis of long-term cortisol secretion 751 patients) through hair assays found no differences between MDD and controls (160). Interestingly, depressed patients with higher levels of cortisol prior to treatment are less likely to benefit from psychological therapy in a metaanalysis of 212 MDD patients investigating cortisol as a predictor of psychological therapy response in depressive disorders (161) (see Table 1).

**Table 1:** Daily HPA measurements in depression.

**References**	**Year**	**Study type**	**Total N**	**N of cases**	**N of control**	**Mode of cortisol collection**	**Findings**
Nikkheslat et al. (152)	2019	Case-Control	218	163	55	Salivary cortisol: Diurnal	The severity of childhood trauma was associated with increased diurnal cortisol levels only in individuals with glucocorticoid resistance
Fischer (154)	2018	Cohort Study	89	37	0	Hair cortisol	No differences were demonstrated in long term cortisol measurements through hair cortisol between those with childhood trauma vs. those without
Mayer et al. (155)	2020	Case-Control	92	56	36	Salivary cortisol and plasma: baseline	There were no baseline cortisol differences in those with MDD-PTSD- child, MDD-PTSD adult and MDD-no trauma
Lopez-Duran et al. (156)	2009	Meta-Analysis	1,332	651	736	Salivary, plasma or urine cortisol: Basal levels	Depressed children and adolescents were found to have greater basal cortisol levels than non-depressed controls
Knorr et al. (157)	2010	Meta-Analysis	2,406	1,354	1,052	Salivary Cortisol	Statistically significant mean difference was found between MDD and healthy individuals in the morning and evening
Aas et al. (162)	2011	Meta-Analysis	18,374	N/A	N/A	Salivary, blood, CSF, urine cortisol	73% of MDD individuals have cortisol values greater than non-depressed individuals. Across all studies, cortisol seems to be elevated by over half an SD unit across depressed individuals. Across all studies, ACTH levels were elevated to a similar degree during MDD
Murri et al. (159)	2013	Meta-Analysis	3,424	727	2,697	Salivary and plasma cortisol	Basal morning cortisol was found to be greater in the morning in MDD patients over 60 years old, morning ACTH levels do not differ between the depressed and healthy group
Psarraki et al. (160)	2020	Meta-Analysis	1,819	751	1,068	Hair cortisol	Long term cortisol secretion measured through hair found no differences between control and MDD
Quidé et al. (163)	2017	Systematic Review and Meta-Analysis	212	212	N/A	Pre-Treatment levels of cortisol inhair, urine, saliva or blood	The higher the basal and post-challenge cortisol levels were before starting psychological therapy, the more symptoms patients experienced at the end of treatment and/or the smaller their symptom change

*A targeted review of key articles and metanalysis showing daily HPA measurements in depression. ACTH, adrenocorticotrophic hormone; CSF, cerebrospinal fluid; MDD, major depressive disorder; PTSD, posttraumatic stress disorder*.

#### No Clear Association Between Cortisol Responses in MDD With ELS

An increase in CAR in those with childhood neglect has been found irrespective of a diagnosis of MDD (164, 165). No correlation between the severity of depression and CAR was shown in those with early life stress (165).

Lower cortisol and ACTH responses were shown following the TSST in children with comorbid MDD and PTSD; however, adults with PTSD commencing in adulthood and those with social anxiety disorder (SAD) showed no differences. When HPA axis feedback was measured through metyrapone challenge, no differences in cortisol levels were found (155). In contrast, a cohort study found positive association between greater depressive symptoms, childhood maltreatment and higher cortisol levels following a TSST (166). Depressed patients with childhood trauma in a different study showed no differences in stress cortisol reactivity following images of child abuse compared to healthy controls (with or without childhood trauma); however, higher reactivity was found in depressed patients with no childhood trauma (167). Conflicting responses to stress tests have been displayed in other meta-analyses. Cortisol reactivity in the morning and afternoon to psychological stress was blunted in a metanalysis of 98 MDD patients compared to controls (168). However, a later meta-analysis of 296 MDD patients showed no significant difference in peak response cortisol levels following social stress (169) (see Table 2).

**Table 2:** HPA responses in depression.

**References**	**Year**	**Study type**	**Total N**	**Number of cases**	**Number of controls**	**Mode of cortisol collection**	**Findings**
Peng et al. (165)	2014	Case- Control	109	58	51	Salivary cortisol: CAR	An increase in CAR in those with childhood neglect irrespective of diagnosis of MDD
Lu et al. (164)	2016	Case- Control	80	35	45	Salivary cortisol: CAR and DST	An increase in CAR in those with childhood neglect irrespective of diagnosis of MDD; The DST responses indicated an increased response in those with MDD and childhood trauma experiences
Mayer et al. (155)	2020	Case- Control	92	56	36	Salivary cortisol: TSST, DST	TSST cortisol responses demonstrated a lowered cortisol and ACTH response in those with MDD and PTSD from childhood vs. controls, however, those with PTSD from adulthood and those with SAD had no differences, when HPA axis feedback was measured through metyrapone challenge, no differences in cortisol levels were found
Cantave et al. (166)	2018	Cohort Study	156	156		Salivary cortisol: CAR	A positive association between higher acute cortisol levels, greater depressive symptoms and childhood maltreatment was demonstrated in the TSST
Suzuki et al. (167)	2014	Case- Control	80	39	41	Salivary cortisol: Images of child abuse	MDD patients with childhood trauma showed no differences in stress cortisol reactivity following images of child abuse compared to healthy controls (with or without childhood trauma); however, higher reactivity was found in patients with no childhood trauma
Watson et al. (170)	2007	Case- Control	68	10	28	Serum cortisol: Dex/CRH test	Those with low levels of emotional neglect showed an enhanced response from the CRH response
Lopez-Duran et al. (156)	2009	Meta-Analysis	926	388	538	Salivary, plasma or urine cortisol: DST	Depressed children and adolescents had higher cortisol production post Dexamethasone suppression test (DST) in contrast to controls. On the other hand, cortisol and ACTH levels post-CRH infusion were non-significant between groups
Aas et al. (162)	2011	Meta-Analysis	1,639	N/A	N/A	Salivary, blood, CSF, urine cortisol	Elevations of cortisol during MDD are greater when the HPA axis is artificially challenged compared to when it is not.
Murri et al. (159)	2013	Meta-Analysis	606	245	361	Salivary and plasma cortisol	No difference was found in the cortisol level post -DST in MDD over 60-year-old adults compared to controls
Burke et al. (168)	2005	Meta-Analysis	196	98	98		MDD patients' stress reactivity cortisol level in the morning and afternoon to psychological stress was blunted in comparison to the healthy counterparts
Ciufolini et al. (169)	2014	Meta-Analysis	800	296	504		No significant difference in peak response cortisol levels post social stress tasks between MDD and control groups
Mokhtari et al. (171)	2012	Meta-Analysis	1,121	670	451		MDD subjects had greater cortisol levels in response to the DEX/CRH test in contrast to healthy controls

*A targeted review of key articles and metanalysis showing HPA responses to awakening, psychological stressors and physiological manipulation. ACTH, adrenocorticotrophic hormone CAR, cortisol awakening response; CRH, corticotrophin releasing hormone; CSF, cerebrospinal fluid; DEX, dexamethasone; DST, dexamethasone suppression test; MDD, major depressive disorder; PTSD, posttraumatic stress disorder; SAD, social anxiety disorder; TSST, Trier social stress test*.

Following dexamethasone suppression an increased cortisol response was found in MDD patients with childhood trauma experiences (164). Interestingly, those with low levels of emotional neglect have shown an enhanced CRH response (170). Depressed children and adolescents (*N* = 388) revealed higher cortisol post DST in a metaanalysis. Conversely, cortisol and ACTH levels post-CRH infusion were non-significant between groups (156). Post DST, higher cortisol and reduced ACTH were found with MDD compared to non-depressed in a metanalysis of 1,639 adults (158). In a metaanalysis of 15 studies examing the role of the dexamethasone /CRH test as potential biomarker for MDD, patients had greater cortisol levels following the test compared to controls (171). No difference was found in the cortisol level post-DST in 245 depressed over 60-year-old adults (159).

#### Elevated Cortisol Turnover and Long-Term Cortisol in BD With ELS

A meta-analysis of 367 BD patients found higher morning cortisol levels were higher in bipolar outpatients and non-manic patients, relative to controls (172). Similarly, another meta-analysis observed higher awakening, morning, afternoon, and evening cortisol for 242 BD patients compared to control (173). While ACTH was raised in the BD group, CRH levels showed no differences between groups. A cohort study measuring cortisol metabolites revealed elevated beta reductase, a measure of cortisol turnover, in BD patients that experienced adverse childhood events such as physical abuse (162). The same group also found elevated long term hair cortisol in those patients with a history of childhood maltreatment (174).

#### Reduced Response Awakening, Stressors and Axis Manipulation in BD With ELS

A blunted CAR was found in a cohort study in BD patients with childhood maltreatment compared to those without (175). BD patients have a higher cortisol level post-DST relative to controls, with further elevation during the manic phase (173). However, the cortisol response to a dexamethasone/CRH test showed enhanced response in BD in those with low levels of emotional neglect (170), similar to those with MDD. Similarly, BD patients with high levels of trauma experienced blunted cortisol responses with associated increased brain activation (the right lingual gyrus and increased functional connectivity between the left amygdala and dorsolateral prefrontal cortex). In contrast, healthy controls with high trauma levels were associated with high cortisol response to stress and decreased brain region activation (163).

### Psychotic Disorders

Psychosis can occur as a primary symptom in disorders such as schizophrenia or manifest as a secondary symptom in other disorders such as MDD and BD (172). Regardless of diagnostic etiology, the core symptom of psychosis involves issues with intact reality testing, resulting in symptoms including hallucinations and/or delusions. The development of psychosis appears to have a strong association with early life events (176).

Schizophrenia is the archetypal psychotic illness characterized by delusions, hallucinations, disorganized speech, disorganized behavior, and negative symptoms. Schizophrenia has a lifetime prevalence of 1% and commonly presents in adolescence and early adulthood (177). It is associated with poor recovery outcomes and reduced life quality expectancy with co-morbidities such as coronary heart disease, stroke, type II diabetes, respiratory diseases, and some cancers (177). A systematic review of 44 studies examining different subtypes of ELS (sexual abuse, physical abuse, emotional abuse, physical neglect, and emotional neglect) concluded that schizophrenia is associated with all forms (178).

#### Increased Baseline Cortisol With Blunted Responses in Psychosis

A meta-analysis examining morning cortisol levels in patients with schizophrenia [44 studies, *n* = 2,613) found a small to medium increase in morning cortisol concentration in patients compared to controls (172). Interestingly, morning samples taken before 8am revealed larger differences than those taken after 8 a.m. Similarly, a higher blood cortisol concentration was shown in a meta-analysis of 911 patients with first-episode psychosis (FEP), most consistent in drug-naive patients of an older presentation (i.e., not adolescents) (179). However, studies examining saliva cortisol concentrations failed to replicate these findings. Interestingly, subgroup analysis of longitudinal studies suggested that cortisol upregulation may be a phenomenon of FEP only with subsequent decrease after antipsychotic treatment.

A meta-analysis of CAR across the psychosis spectrum [11 studies, *n* = 879) found that the response was lessened in patients with psychosis compared to healthy controls (180). Subgroup analysis found a flattened CAR in patients with schizophrenia and FEP, but not in individuals with at-risk-mental states, leading the authors to suggest that the response may be a marker for transition risk. In a recent meta-analysis of cortisol reactivity to psychological stress in patients with schizophrenia (4 studies, *n* = 180), patients demonstrated a blunted response compared to controls, with males in particular revealing lower cortisol AUCi and AUCg following stressors (181). The authors acknowledge the possibility of publication bias in favor for the male blunted reactivity in schizophrenia.

#### Increased Turnover With Less Blunted Responses in Psychosis With ELS

Using a stable marker of cortisol concentration over several months (hair), cortisol was elevated in a study of patients with schizophrenia (*n* = 28) who had a history of childhood maltreatment (174). Similarly, elevated cortisol metabolism (through urinary analysis of cortisol metabolites: e.g., urinary free cortisol/cortisone, allo-tetrahydrocortisol, tetrahydrocortisol, and tetrahydrocortisone) was found in schizophrenia patients (*n* = 63) with childhood trauma compared to those without trauma (162).

In a study of 9 to 12 year old children with either clinical high risk of psychosis (*n* = 33), a family history of psychosis (*n* = 22), or typically developing children (*n* = 40), no association was found between the CAR and the numbers of negative life events in any group (182). However, in the family history group, CAR was positively correlated with distress experienced in relation to negative life events at the time of the event and with the level of distress experienced currently. In contrast, among typically developing children, CAR values were negatively correlated with distress experienced at the time of the negative life event. Schizophrenia spectrum patients (*n* = 25) demonstrated a blunted response to a psychosocial stressor (a modified Triers Social Stress tasking involving public speaking) compared to controls (*n* = 25) (183) with patients with a history of more emotional abuse showing a response closer to controls. Similarly, in a study of emotional brain function (functional magnetic resonance imaging while performing an emotional face-matching task) and cortisol reactivity in patients with schizophrenia (*n* = 40) and controls (*n* = 34) found that cortisol concentrations reduced in patients and increased in controls following the task (163). In those exposed to high levels of trauma, higher post scan cortisol was associated with region activation in the schizophrenia cohort. As such, ELS and psychosis are associated with high ambient cortisol and less flattened responses.

## Conclusion

The relationship between early life stress (ELS), Hypothalamic Pituitary Adrenal (HPA) axis activity and psychiatric illnesses is complex. The development of the brain during childhood involves sequential and heirarchical development of brain regions and is susceptible to biological and psychological insults, particularly during developmentally sensitive periods. Toxic stress from childhood adversity may result in sympathetic hyperarousal through sustained allostatic load along the hodological associations of the HPA axis. Such connections include limbic structures involved in memory, behavior and emotion such as the hippocampus, amygdala and the medial prefrontal cortex. Inconsistencies exist in the literature regarding the hyperactivation of the HPA axis in adolescents who experienced childhood trauma. As such, it is thought that HPA axis changes may specific to the trauma subtype being studied, with further research needed in the domain.

Our targeted review of the literature surrounding mood disorders and psychosis suggests that cortisol concentration is raised across psychiatric disorders. However, both bipolar disorder (BD) and psychosis is associated with more attenuated HPA responses to awakening (the cortisol awakening response), psychologicial stressors (e.g., the Trier Social Stress Test) and physiological manipulation (e.g., dexamethasone supression) in those patients who have experienced early life stress compared to those who have not. These reduced cortisol responses in BD and psychotic patients exist on a common background of increased long term cortisol and cortisol turnover. This suggests a hyperfunctioning HPA axis with little reserve remaining for a robust cortisol response to extra stress. The evidence from our targeted review for a similar effect in depression (MDD) is equivocal, despite the signficantly larger number of studies examining early life stress and HPA changes in MDD.

Psychosis and BD share a number of commonalities in their biological basis. Psychosis is often a feature of mania in Type 1 BD (a diagnosis of mania requires either psychosis or hospitalization) (184). Common susceptibility genes for both disorders have also been identified. The first of such commonalities identified included zinc finger–binding protein 804A gene (ZNF804A) on chromosome 2q32 (185). Since then, additional areas of interest such as 1p36, 1q43, 4p14 and, of particular note, 15q26 have been identified (186). Schizoaffective disorder is another notable disorder under the heading of psychosis, often described as the intermediate of BD and schizophrenia, involving both affective and psychotic tendencies. Further genetic linkages have been implicated as common to all three disorders, of particular note COMT located in the 22q11 region (187) as well as genome wide significance at 1q42 (188). COMT is involved in the metabolism of catecholamines including noradrenaline and adrenaline (neurotransmitters known to be involved in acute stress). In contrast, loss of function polymorphisms in tryptophan hydroxylase 2 (TPH-2) have been identified in MDD but have been shown not to be implicated in the pathogenesis of BD (189) or SZ (190). TPH2 is a key enzyme in formation of serotonin, with dysfunction of the TPH-2 associated with shunting of tryptophan toward kynurenine, a key pathway at the interface of inflammation and the stress response (191). This may be a mechanism for different cortisol profiles between MDD and BD/psychosis.

Structural abnormalities common to both BD and schizophrenia have also been observed on neuroimaging. Diffusion studies of the uncinate fasciculus (79) (connecting the amygdala with the medial- and orbitofrontal cortices) and anterior and posterior thalamic radiations (192) have shown decreased fractional anistrophy (a marker of white matter microstructural abnormality) in both BD and schizophrenia. These connections are known to be involved in the stress response.

In conclusion, the effect of the early life stress on the developing brain is highly complex and dependent on unique characteristics such as individual vulnerability, developmental sensitivity window, stressor type and duration. The suggestion of a common HPA profile in both BD and psychosis but not depression from our targeted review needs verification with large carefully conducted robust metaanalyses of daily cortisol concentrations and cortisol responses across these disorders.

## Author Contributions

AN, FM, DC, KR, AG, and ER: writing, editing, and formatting manuscript. VS, LK, AO, and MH: proofreading and editing. DR and MC: leading the group, writing, editing, and formatting manuscript. All authors contributed to the article and approved the submitted version.

## Funding

The work was funded by iHEAR study - MC.

## Conflict of Interest

The authors declare that the research was conducted in the absence of any commercial or financial relationships that could be construed as a potential conflict of interest.

## Publisher's Note

All claims expressed in this article are solely those of the authors and do not necessarily represent those of their affiliated organizations, or those of the publisher, the editors and the reviewers. Any product that may be evaluated in this article, or claim that may be made by its manufacturer, is not guaranteed or endorsed by the publisher.

## References

[1] Arborelius L Owens M Plotsky P Nemeroff CB. The role of corticotropin-releasing factor in depression and anxiety disorders. J Endocrinol. (1999) 160:1–12. 10.1677/joe.0.1600001 9854171

[2] Heim C Newport DJ Mletzko T Miller AH Nemeroff CB. The link between childhood trauma and depression: insights from HPA axis studies in humans. Psychoneuroendocrinology. (2008) 33:693–710. 10.1016/j.psyneuen.2008.03.008 18602762

[3] Ivanov I Yehuda R Greenblatt E Davidow J Makotkine I Alfi L. The effect of trauma on stress reactivity in aggressive youth. Psychiatry Res. (2011) 189:396–402. 10.1016/j.psychres.2011.05.046 21684014

[4] De Bellis MD Zisk A. The biological effects of childhood trauma. Child Adolesc Psychiatr Clin. (2014) 23:185–222. 10.1016/j.chc.2014.01.002 24656576 PMC3968319

[5] Middlebrooks JS Audage NC. The Effects of Childhood Stress on Health Across the Lifespan, Centers for Disease Control and Prevention. Atlanta, GA (2008). 10.1037/e721332007-001

[6] Felitti VJ Anda RF Nordenberg D Williamson DF Spitz AM Edwards V. Relationship of childhood abuse and household dysfunction to many of the leading causes of death in adults: The Adverse Childhood Experiences (ACE) Study. Am J Prev Med. (1998) 14:245–58. 10.1016/S0749-3797(98)00017-8 9635069

[7] Bernstein DP Fink L Handelsman L Foote J. Childhood Trauma Questionnaire. Assessment of Family Violence: A Handbook for Researchers and Practitioners. Orlando, FL: Psychological Corporation (1998).

[8] Bremner JD Bolus R Mayer EA. Psychometric properties of the early trauma inventory–self report. J Nerv Ment Dis. (2007) 195:211. 10.1097/01.nmd.0000243824.84651.6c 17468680 PMC3229091

[9] Bethell CD Carle A Hudziak J Gombojav N Powers K Wade R. Methods to assess adverse childhood experiences of children and families: toward approaches to promote child well-being in policy and practice. Acad Pediatr. (2017) 17:S51–69. 10.1016/j.acap.2017.04.161 28865661 PMC6035880

[10] May-Chahal C Cawson P. Measuring child maltreatment in the United Kingdom: a study of the prevalence of child abuse and neglect. Child Abuse Negl. (2005) 29:969–84. 10.1016/j.chiabu.2004.05.009 16165212

[11] Afifi TO MacMillan HL Boyle M Taillieu T Cheung K Sareen J. Child abuse and mental disorders in Canada. CMAJ. (2014) 186:E324–32. 10.1503/cmaj.131792 24756625 PMC4050024

[12] Mathews B Pacella R Dunne MP Simunovic M Marston C. Improving measurement of child abuse and neglect: a systematic review and analysis of national prevalence studies. PLoS ONE. (2020) 15:e0227884. 10.1371/journal.pone.0227884 31990913 PMC6986759

[13] Edwards VJ Holden GW Felitti VJ Anda RF. Relationship between multiple forms of childhood maltreatment and adult mental health in community respondents: results from the adverse childhood experiences study. Am J Psychiatry. (2003) 160:1453–60. 10.1176/appi.ajp.160.8.1453 12900308

[14] Schäfer I Fisher HL. Childhood trauma and psychosis-what is the evidence? Dialogues Clin Neurosci. (2011) 13:360. 10.31887/DCNS.2011.13.2/ischaefer 22033827 PMC3182006

[15] Lewis SJ Arseneault L Caspi A Fisher HL Matthews T Moffitt TE. The epidemiology of trauma and post-traumatic stress disorder in a representative cohort of young people in England and Wales. Lancet Psychiatry. (2019) 6:247–56. 10.1016/S2215-0366(19)30031-8 30798897 PMC6384243

[16] Kessler RC McLaughlin KA Green JG Gruber MJ Sampson NA Zaslavsky AM. Childhood adversities and adult psychopathology in the WHO World Mental Health Surveys. Br J Psychiatry. (2010) 197:378–85. 10.1192/bjp.bp.110.080499 21037215 PMC2966503

[17] Perry BD. Childhood experience and the expression of genetic potential: what childhood neglect tells us about nature and nurture. Brain Mind. (2002) 3:79–100. 10.1023/A:1016557824657

[18] Perry BD. Examining child maltreatment through a neurodevelopmental lens: clinical applications of the neurosequential model of therapeutics. J Loss Trauma. (2009) 14:240–55. 10.1080/15325020903004350

[19] Ismail FY Fatemi A Johnston MV. Cerebral plasticity: windows of opportunity in the developing brain. Eur J Paediatr Neurol. (2017) 21:23–48. 10.1016/j.ejpn.2016.07.007 27567276

[20] Knudsen EI. Sensitive periods in the development of the brain and behavior. J Cogn Neurosci. (2004) 16:1412–25. 10.1162/0898929042304796 15509387

[21] Johnson MH. Sensitive periods in functional brain development: problems and prospects. Dev Psychobiol. (2005) 46:287–92. 10.1002/dev.20057 15772965

[22] Knudsen E. Mechanisms of experience-dependent plasticity in the auditory localization pathway of the barn owl. J Comp Physiol A. (1999) 185:305–21. 10.1007/s003590050391 10555267

[23] Thomas MS Johnson MH. New advances in understanding sensitive periods in brain development. Curr Dir Psychol Sci. (2008) 17:1–5. 10.1111/j.1467-8721.2008.00537.x

[24] Thomas MS Johnson MH. The computational modeling of sensitive periods. Dev Psychobiol. (2006) 48:337. 10.1002/dev.20134 16617466

[25] Callaghan BL Tottenham N. The stress acceleration hypothesis: effects of early-life adversity on emotion circuits and behavior. Curr Opin Behav Sci. (2016) 7:76–81. 10.1016/j.cobeha.2015.11.018 29644262 PMC5890821

[26] McLaughlin KA Weissman D Bitrán D. Childhood adversity and neural development: a systematic review. Annu Rev Dev Psychol. (2019) 1:277–312. 10.1146/annurev-devpsych-121318-084950 32455344 PMC7243625

[27] Nardou R Lewis EM Rothhaas R Xu R Yang A Boyden E. Oxytocin-dependent reopening of a social reward learning critical period with MDMA. Nature. (2019) 569:116–20. 10.1038/s41586-019-1075-9 30944474

[28] Sharif MH Talebnejad MR Rastegar K Khalili MR Nowroozzadeh MH. Oral fluoxetine in the management of amblyopic patients aged between 10 and 40 years old: a randomized clinical trial. Eye. (2019) 33:1060–7. 10.1038/s41433-019-0360-z 30783259 PMC6707246

[29] Boldrini M Underwood MD Hen R Rosoklija GB Dwork AJ John Mann J. Antidepressants increase neural progenitor cells in the human hippocampus. Neuropsychopharmacology. (2009) 34:2376–89. 10.1038/npp.2009.75 19606083 PMC2743790

[30] Dringenberg HC Branfield Day LR Choi DH. Chronic fluoxetine treatment suppresses plasticity (long-term potentiation) in the mature rodent primary auditory cortex in vivo. Neural Plast. (2014) 2014:571285. 10.1155/2014/571285 24719772 PMC3956292

[31] Gervain J Vines BW Chen LM Seo RJ Hensch TK Werker JF. Valproate reopens critical-period learning of absolute pitch. Front Syst Neurosci. (2013) 7:102. 10.3389/fnsys.2013.00102 24348349 PMC3848041

[32] Perry BD Pollard RA Blakley TL Baker WL Vigilante D. Childhood trauma, the neurobiology of adaptation, and “use-dependent” development of the brain: how “states” become “traits”. Infant Ment Health J. (1995) 16:271–91.

[33] Kim HG Cheon EJ Bai DS Lee YH Koo BH. Stress and heart rate variability: a meta-analysis and review of the literature. Psychiatry Investig. (2018) 15:235. 10.30773/pi.2017.08.17 29486547 PMC5900369

[34] Weber DA Reynolds CR. Clinical perspectives on neurobiological effects of psychological trauma. Neuropsychol Rev. (2004) 14:115–29. 10.1023/B:NERV.0000028082.13778.14 15264712

[35] Brunson K Avishai-Eliner S Hatalski C Baram T. Neurobiology of the stress response early in life: evolution of a concept and the role of corticotropin releasing hormone. Mol Psychiatry. (2001) 6:647–56. 10.1038/sj.mp.4000942 11673792 PMC3100722

[36] Heim C Nemeroff CB. The role of childhood trauma in the neurobiology of mood and anxiety disorders: preclinical and clinical studies. Biol Psychiatry. (2001) 49:1023–39. 10.1016/S0006-3223(01)01157-X 11430844

[37] De Bellis MD. The psychobiology of neglect. Child Maltreat. (2005) 10:150–72. 10.1177/1077559505275116 15798010

[38] Wilson KR Hansen DJ Li M. The traumatic stress response in child maltreatment and resultant neuropsychological effects. Aggress Violent Behav. (2011) 16:87–97. 10.1016/j.avb.2010.12.007

[39] Spiga F Walker JJ Terry JR Lightman SL. HPA axis-rhythms. Compr Physiol. (2011) 4:1273–98. 10.1002/cphy.c140003 24944037

[40] Pruessner JC Hellhammer DH Kirschbaum C. Burnout, perceived stress, and cortisol responses to awakening. Psychosom Med. (1999) 61:197–204. 10.1097/00006842-199903000-00012 10204973

[41] Miller GE Chen E Zhou ES. If it goes up, must it come down? Chronic stress and the hypothalamic-pituitary-adrenocortical axis in humans. Psychol Bull. (2007) 133:25–45. 10.1037/0033-2909.133.1.25 17201569

[42] O'Connor DB Branley-Bell D Green JA Ferguson E O'Carroll RE O'Connor RC. Effects of childhood trauma, daily stress, and emotions on daily cortisol levels in individuals vulnerable to suicide. J Abnorm Psychol. (2020) 129:92. 10.1037/abn0000482 31657598

[43] Fries E Hesse J Hellhammer J Hellhammer DH. A new view on hypocortisolism. Psychoneuroendocrinology. (2005) 30:1010–6. 10.1016/j.psyneuen.2005.04.006 15950390

[44] McEwen BS. Stress, adaptation, and disease: allostasis and allostatic load. Ann N Y Acad Sci. (1998) 840:33–44. 10.1111/j.1749-6632.1998.tb09546.x 9629234

[45] McEwen BS Wingfield JC. The concept of allostasis in biology and biomedicine. Horm Behav. (2003) 43:2–15. 10.1016/S0018-506X(02)00024-7 12614627

[46] MacMaster FP Kusumakar V. MRI study of the pituitary gland in adolescent depression. J Psychiatr Res. (2004) 38:231–6. 10.1016/j.jpsychires.2003.11.001 15003427

[47] Pariante CM Dazzan P Danese A Morgan KD Brudaglio F Morgan C. Increased pituitary volume in antipsychotic-free and antipsychotic-treated patients of the aesop first-onset psychosis study. Neuropsychopharmacology. (2005) 30:1923–31. 10.1038/sj.npp.1300766 15956995

[48] Eker C Ovali GY Ozan E Eker OD Kitis O Coburn K. No pituitary gland volume change in medication-free depressed patients. Prog Neuropsychopharmacol Biol Psychiatry. (2008) 32:1628–32. 10.1016/j.pnpbp.2008.05.023 18573301

[49] Lorenzetti V Allen NB Fornito A Pantelis C De Plato G Ang A. Pituitary gland volume in currently depressed and remitted depressed patients. Psychiatry Res. (2009) 172:55–60. 10.1016/j.pscychresns.2008.06.006 19239986

[50] MacMaster FP Russell A Mirza Y Keshavan MS Taormina SP Bhandari R. Pituitary volume in treatment-naive pediatric major depressive disorder. Biol Psychiatry. (2006) 60:862–6. 10.1016/j.biopsych.2006.04.013 16876142

[51] Delvecchio G Mandolini GM Perlini C Barillari M Marinelli V Ruggeri M. Pituitary gland shrinkage in bipolar disorder: the role of gender. Compr Psychiatry. (2018) 82:95–9. 10.1016/j.comppsych.2018.01.014 29454165

[52] Cousins DA Moore PB Watson S Harrison L Ferrier IN Young AH. Pituitary volume and third ventricle width in euthymic patients with bipolar disorder. Psychoneuroendocrinology. (2010) 35:1074–81. 10.1016/j.psyneuen.2010.01.008 20171783

[53] Nordholm D Krogh J Mondelli V Dazzan P Pariante C Nordentoft M. Pituitary gland volume in patients with schizophrenia, subjects at ultra high-risk of developing psychosis and healthy controls: a systematic review and meta-analysis. Psychoneuroendocrinology. (2013) 38:2394–404. 10.1016/j.psyneuen.2013.06.030 23890984

[54] Borges S Gayer-Anderson C Mondelli V. A systematic review of the activity of the hypothalamic–pituitary–adrenal axis in first episode psychosis. Psychoneuroendocrinology. (2013) 38:603–11. 10.1016/j.psyneuen.2012.12.025 23369532

[55] Aiello G Horowitz M Hepgul N Pariante CM Mondelli V. Stress abnormalities in individuals at risk for psychosis: a review of studies in subjects with familial risk or with “at risk” mental state. Psychoneuroendocrinology. (2012) 37:1600–13. 10.1016/j.psyneuen.2012.05.003 22663896

[56] Saunders TS Mondelli V Cullen AE. Pituitary volume in individuals at elevated risk for psychosis: a systematic review and meta-analysis. Schizophr Res. (2019) 213:23–31. 10.1016/j.schres.2018.12.026 30600112

[57] Pariante CM Vassilopoulou K Velakoulis D Phillips L Soulsby B Wood SJ. Pituitary volume in psychosis. Br J Psychiatry. (2004) 185:5–10. 10.1192/bjp.185.1.5 15231549

[58] Upadhyaya AR El-Sheikh R MacMaster FP Diwadkar VA Keshavan MS. Pituitary volume in neuroleptic-naive schizophrenia: a structural MRI study. Schizophr Res. (2007) 90:266–73. 10.1016/j.schres.2006.09.033 17187962

[59] Weininger J Roman E Tierney P Barry D Gallagher H Murphy P. Papez's forgotten tract: 80 years of unreconciled findings concerning the thalamocingulate tract. Front Neuroanat. (2019) 13:e14. 10.3389/fnana.2019.00014 30833890 PMC6388660

[60] Nolan M Roman E Nasa A Levins KJ O'Hanlon E O'Keane V. Hippocampal and amygdalar volume changes in major depressive disorder: a targeted review and focus on stress. Chronic Stress. (2020) 4:2470547020944553. 10.1177/2470547020944553 33015518 PMC7513405

[61] Roddy D Kelly JR Farrell C Doolin K Roman E Nasa A. Amygdala substructure volumes in major depressive disorder. Neuroimage Clin. (2021) 31:102781. 10.1016/j.nicl.2021.102781 34384996 PMC8361319

[62] Roddy D O'Keane V. Cornu ammonis changes are at the core of hippocampal pathology in depression. Chronic Stress. (2019) 3:2470547019849376. 10.1177/2470547019849376 32440594 PMC7219935

[63] Herman JP Cullinan WE. Neurocircuitry of stress: central control of the hypothalamo–pituitary–adrenocortical axis. Trends Neurosci. (1997) 20:78–84. 10.1016/S0166-2236(96)10069-2 9023876

[64] Radley JJ Sawchenko PE. A common substrate for prefrontal and hippocampal inhibition of the neuroendocrine stress response. J Neurosci. (2011) 31:9683–95. 10.1523/JNEUROSCI.6040-10.2011 21715634 PMC3197245

[65] Herman J. Neural control of chronic stress adaptation. Front Behav Neurosci. (2013) 7:61. 10.3389/fnbeh.2013.00061 23964212 PMC3737713

[66] Feldman S Conforti N Itzik A Weidenfeld J. Differential effect of amygdaloid lesions on CRF-41, ACTH and corticosterone responses following neural stimuli. Brain Res. (1994) 658:21–6. 10.1016/S0006-8993(09)90005-1 7834344

[67] Van de Kar LD Piechowski RA Rittenhouse PA Gray TS. Amygdaloid lesions: differential effect on conditioned stress and immobilization-induced increases in corticosterone and renin secretion. Neuroendocrinology. (1991) 54:89–95. 10.1159/000125856 1766554

[68] Prewitt CM Herman JP. Hypothalamo-pituitary-adrenocortical regulation following lesions of the central nucleus of the amygdala. Stress. (1997) 1:263–79. 10.3109/10253899709013746 9787250

[69] Flandreau EI Ressler KJ Owens MJ Nemeroff CB. Chronic overexpression of corticotropin-releasing factor from the central amygdala produces HPA axis hyperactivity and behavioral anxiety associated with gene-expression changes in the hippocampus and paraventricular nucleus of the hypothalamus. Psychoneuroendocrinology. (2012) 37:27–38. 10.1016/j.psyneuen.2011.04.014 21616602 PMC3164918

[70] Weidenfeld J Ovadia H. The Role of the Amygdala in Regulating the Hypothalamic-Pituitary-Adrenal axis. The Amygdala: Where Emotions Shape Perception, Learning and Memories. In: Ferry B, editor. London: IntechOpen (2017). pp. 173–186.

[71] Cullinan WE Herman JP Battaglia DF Akil H Watson S. Pattern and time course of immediate early gene expression in rat brain following acute stress. Neuroscience. (1995) 64:477–505. 10.1016/0306-4522(94)00355-9 7700534

[72] Figueiredo HF Bruestle A Bodie B Dolgas CM Herman JP. The medial prefrontal cortex differentially regulates stress-induced c-fos expression in the forebrain depending on type of stressor. Eur J Neurosci. (2003) 18:2357–64. 10.1046/j.1460-9568.2003.02932.x 14622198

[73] Coplan JD Fathy HM Jackowski AP Tang CY Perera TD Mathew SJ. Early life stress and macaque amygdala hypertrophy: preliminary evidence for a role for the serotonin transporter gene. Front Behav Neurosci. (2014) 8:342. 10.3389/fnbeh.2014.00342 25339875 PMC4186477

[74] Tottenham N Hare TA Quinn BT McCarry TW Nurse M Gilhooly T. Prolonged institutional rearing is associated with atypically large amygdala volume and difficulties in emotion regulation. Dev Sci. (2010) 13:46–61. 10.1111/j.1467-7687.2009.00852.x 20121862 PMC2817950

[75] Lupien SJ Parent S Evans AC Tremblay RE Zelazo PD Corbo V. Larger amygdala but no change in hippocampal volume in 10-year-old children exposed to maternal depressive symptomatology since birth. Proc Nat Acad Sci USA. (2011) 108:14324–9. 10.1073/pnas.1105371108 21844357 PMC3161565

[76] Woon FL Hedges DW. Hippocampal and amygdala volumes in children and adults with childhood maltreatment-related posttraumatic stress disorder: a meta-analysis. Hippocampus. (2008) 18:729–36. 10.1002/hipo.20437 18446827

[77] Bremner JD Randall P Vermetten E Staib L Bronen RA Mazure C. Magnetic resonance imaging-based measurement of hippocampal volume in posttraumatic stress disorder related to childhood physical and sexual abuse—a preliminary report. Biol Psychiatry. (1997) 41:23–32. 10.1016/S0006-3223(96)00162-X 8988792 PMC3229101

[78] Usher J Leucht S Falkai P Scherk H. Correlation between amygdala volume and age in bipolar disorder—a systematic review and meta-analysis of structural MRI studies. Psychiatry Res Neuroimaging. (2010) 182:1–8. 10.1016/j.pscychresns.2009.09.004 20226638

[79] Ho NF Chong PLH Lee DR Chew QH Chen G Sim K. The amygdala in schizophrenia and bipolar disorder: a synthesis of structural MRI, diffusion tensor imaging, and resting-state functional connectivity findings. Harv Rev Psychiatry. (2019) 27:150–64. 10.1097/HRP.0000000000000207 31082993

[80] Ganzola R Maziade M Duchesne S. Hippocampus and amygdala volumes in children and young adults at high-risk of schizophrenia: research synthesis. Schizophr Res. (2014) 156:76–86. 10.1016/j.schres.2014.03.030 24794883

[81] Mahon PB Eldridge H Crocker B Gindes H Postell E King S. An MRI study of amygdala in schizophrenia and psychotic bipolar disorder. Schizophr Res. (2012) 138:188–91. 10.1016/j.schres.2012.04.005 22559949 PMC3372630

[82] Bois C Levita L Ripp I Owens DC Johnstone EC Whalley HC. Hippocampal, amygdala and nucleus accumbens volume in first-episode schizophrenia patients and individuals at high familial risk: a cross-sectional comparison. Schizophr Res. (2015) 165:45–51. 10.1016/j.schres.2015.03.024 25864953

[83] Witthaus H Mendes U Brüne M Özgürdal S Bohner G Gudlowski Y. Hippocampal subdivision and amygdalar volumes in patients in an at-risk mental state for schizophrenia. J Psychiatry Neurosci. (2010) 35:33. 10.1503/jpn.090013 20040244 PMC2799502

[84] Tanskanen P Veijola JM Piippo UK Haapea M Miettunen JA Pyhtinen J. Hippocampus and amygdala volumes in schizophrenia and other psychoses in the Northern Finland 1966 birth cohort. Schizophr Res. (2005) 75:283–94. 10.1016/j.schres.2004.09.022 15885519

[85] Goldman AL Pezawas L Mattay VS Fischl B Verchinski BA Zoltick B. Heritability of brain morphology related to schizophrenia: a large-scale automated magnetic resonance imaging segmentation study. Biol Psychiatry. (2008) 63:475–83. 10.1016/j.biopsych.2007.06.006 17727823

[86] Barth C Nerland S de Lange AMG Wortinger LA Hilland E Andreassen OA. *In vivo* amygdala nuclei volumes in schizophrenia and bipolar disorders. Schizophr Bull. (2021) 47:1431–41. 10.1101/2020.09.30.20204602 33479754 PMC8379533

[87] Hoy K Barrett S Shannon C Campbell C Watson D Rushe T. Childhood trauma and hippocampal and amygdalar volumes in first-episode psychosis. Schizophr Bull. (2012) 38:1162–9. 10.1093/schbul/sbr085 21799213 PMC3494041

[88] Dunn J Orr S. Differential plasma corticosterone responses to hippocampal stimulation. Exp Brain Res. (1984) 54:1–6. 10.1007/BF00235813 6321219

[89] Magarinos A Somoza G De Nicola A. Glucocorticoid negative feedback and glucocorticoid receptors after hippocampectomy in rats. Horm Metab Res. (1987) 19:105–9. 10.1055/s-2007-1011753 3570145

[90] Sapolsky RM Krey LC McEwen BS. Glucocorticoid-sensitive hippocampal neurons are involved in terminating the adrenocortical stress response. Proc Nat Acad Sci USA. (1984) 81:6174–7. 10.1073/pnas.81.19.6174 6592609 PMC391882

[91] Kim EJ Pellman B Kim JJ. Stress effects on the hippocampus: a critical review. Learn Mem. (2015) 22:411–6. 10.1101/lm.037291.114 26286651 PMC4561403

[92] Lajud N Torner L. Early life stress and hippocampal neurogenesis in the neonate: sexual dimorphism, long term consequences and possible mediators. Front Mol Neurosci. (2015) 8:e3. 10.3389/fnmol.2015.00003 25741234 PMC4327304

[93] Stein MB Koverola C Hanna C Torchia M McClarty B. Hippocampal volume in women victimized by childhood sexual abuse. Psychol Med. (1997) 27:951–9. 10.1017/S0033291797005242 9234472

[94] Lee T Jarome T Li SJ Kim JJ Helmstetter FJ. Chronic stress selectively reduces hippocampal volume in rats: a longitudinal MRI study. Neuroreport. (2009) 20:1554. 10.1097/WNR.0b013e328332bb09 19858767 PMC2783199

[95] Drago T O'Regan PW Welaratne I Rooney S O'Callaghan A Malkit M. A comprehensive regional neurochemical theory in depression: a protocol for the systematic review and meta-analysis of 1H-MRS studies in major depressive disorder. Syst Rev. (2018) 7:1–6. 10.1186/s13643-018-0830-6 30309391 PMC6182786

[96] Levins KJ Drago T Roman E Martin A King R Murphy P. Magnetic resonance spectroscopy across chronic pain disorders: a systematic review protocol synthesising anatomical and metabolite findings in chronic pain patients. Syst Rev. (2019) 8:1–7. 10.1186/s13643-019-1256-5 31882014 PMC6935150

[97] Yildiz-Yesiloglu A Ankerst DP. Review of 1H magnetic resonance spectroscopy findings in major depressive disorder: a meta-analysis. Psychiatry Res Neuroimaging. (2006) 147:1–25. 10.1016/j.pscychresns.2005.12.004 16806850

[98] Van Rooij S Kennis M Sjouwerman R Van Den Heuvel M Kahn R Geuze E. Smaller hippocampal volume as a vulnerability factor for the persistence of post-traumatic stress disorder. Psychol Med. (2015) 45:2737–46. 10.1017/S0033291715000707 25936409

[99] Roddy DW Farrell C Doolin K Roman E Tozzi L Frodl T. The hippocampus in depression: more than the sum of its parts? advanced hippocampal substructure segmentation in depression. Biol Psychiatry. (2018) 85:487–97. 10.1016/j.biopsych.2018.08.021 30528746

[100] Buddeke J Kooistra M Zuithoff NP Gerritsen L Biessels GJ van der Graaf Y. Hippocampal volume and the course of depressive symptoms over eight years of follow-up. Acta Psychiatr Scand. (2017) 135:78–86. 10.1111/acps.12662 27800603

[101] Barch DM Tillman R Kelly D Whalen D Gilbert K Luby JL. Hippocampal volume and depression among young children. Psychiatry Res Neuroimaging. (2019) 288:21–8. 10.1016/j.pscychresns.2019.04.012 31071541 PMC6550342

[102] Maller J Broadhouse K Rush A Gordon E Koslow S Grieve S. Increased hippocampal tail volume predicts depression status and remission to anti-depressant medications in major depression. Mol Psychiatry. (2018) 23:1737–44. 10.1038/mp.2017.224 29133948

[103] Weniger G Lange C Irle E. Abnormal size of the amygdala predicts impaired emotional memory in major depressive disorder. J Affect Disord. (2006) 94:219–29. 10.1016/j.jad.2006.04.017 16740316

[104] Frey BN Andreazza AC Nery FG Martins MR Quevedo J Soares JC. The role of hippocampus in the pathophysiology of bipolar disorder. Behav Pharmacol. (2007) 18:419–30. 10.1097/FBP.0b013e3282df3cde 17762510

[105] Hauser P Matochik J Altshuler LL Denicoff KD Conrad A Li X. MRI-based measurements of temporal lobe and ventricular structures in patients with bipolar I and bipolar II disorders. J Affect Disord. (2000) 60:25–32. 10.1016/S0165-0327(99)00154-8 10940444

[106] Altshuler LL Bartzokis G Grieder T Curran J Jimenez T Leight K. An MRI study of temporal lobe structures in men with bipolar disorder or schizophrenia. Biol Psychiatry. (2000) 48:147–62. 10.1016/S0006-3223(00)00836-2 10903411

[107] Chepenik LG Wang F Spencer L Spann M Kalmar JH Womer F. Structure–function associations in hippocampus in bipolar disorder. Biol Psychol. (2012) 90:18–22. 10.1016/j.biopsycho.2012.01.008 22342942 PMC3319637

[108] Haukvik UK Tamnes CK Söderman E Agartz I. Neuroimaging hippocampal subfields in schizophrenia and bipolar disorder: a systematic review and meta-analysis. J Psychiatr Res. (2018) 104:217–26. 10.1016/j.jpsychires.2018.08.012 30107268

[109] Han KM Kim A Kang W Kang Y Kang J Won E. Hippocampal subfield volumes in major depressive disorder and bipolar disorder. Eur Psychiatry. (2019) 57:70–7. 10.1016/j.eurpsy.2019.01.016 30721801

[110] Janiri D Sani G De Rossi P Piras F Banaj N Ciullo V. Hippocampal subfield volumes and childhood trauma in bipolar disorders. J Affect Disord. (2019) 253:35–43. 10.1016/j.jad.2019.04.071 31022627

[111] Janiri D Sani G Rossi PD Piras F Iorio M Banaj N. Amygdala and hippocampus volumes are differently affected by childhood trauma in patients with bipolar disorders and healthy controls. Bipolar Disord. (2017) 19:353–62. 10.1111/bdi.12516 28699182

[112] Kalmady SV Shivakumar V Arasappa R Subramaniam A Gautham S Venkatasubramanian G. Clinical correlates of hippocampus volume and shape in antipsychotic-naïve schizophrenia. Psychiatry Res Neuroimaging. (2017) 263:93–102. 10.1016/j.pscychresns.2017.03.014 28371658

[113] Haukvik UK Hartberg CB Agartz I. Schizophrenia–what does structural MRI show? Tidsskr Nor Legeforen. (2013) 133:850–3. 10.4045/tidsskr.12.1084 23612107

[114] Sauras R Keymer A Alonso-Solis A Díaz A Molins C Nuñez F. Volumetric and morphological characteristics of the hippocampus are associated with progression to schizophrenia in patients with first-episode psychosis. Eur Psychiatry. (2017) 45:1–5. 10.1016/j.eurpsy.2017.06.006 28728089

[115] Watson DR Bai F Barrett SL Turkington A Rushe TM Mulholland CC. Structural changes in the hippocampus and amygdala at first episode of psychosis. Brain Imaging Behav. (2012) 6:49–60. 10.1007/s11682-011-9141-4 22045236

[116] Adriano F Caltagirone C Spalletta G. Hippocampal volume reduction in first-episode and chronic schizophrenia: a review and meta-analysis. Neuroscientist. (2012) 18:180–200. 10.1177/1073858410395147 21531988

[117] Nasa A Mosley O Roman E Kelliher A Gaughan C Levins KJ. MRI volumetric changes in hippocampal subfields in psychosis: a protocol for a systematic review and meta-analysis. Syst Rev. (2022) 11:44. 10.1186/s13643-022-01916-5 35292116 PMC8925181

[118] Carrion VG Wong SS. Can traumatic stress alter the brain? Understanding the implications of early trauma on brain development and learning. J Adolesc Health. (2012) 51:S23–8. 10.1016/j.jadohealth.2012.04.010 22794529

[119] Ancelin ML Carriere I Artero S Maller JJ Meslin C Dupuy AM. Structural brain changes with lifetime trauma and re-experiencing symptoms is 5-HTTLPR genotype-dependent. Eur J Psychotraumatol. (2020) 11:1733247. 10.1080/20008198.2020.1733247 32194924 PMC7067154

[120] van Harmelen AL van Tol MJ van der Wee NJ Veltman DJ Aleman A Spinhoven P. Reduced medial prefrontal cortex volume in adults reporting childhood emotional maltreatment. Biol Psychiatry. (2010) 68:832–8. 10.1016/j.biopsych.2010.06.011 20692648

[121] Underwood MD Bakalian MJ Escobar T Kassir S Mann JJ Arango V. Early-life adversity, but not suicide, is associated with less prefrontal cortex gray matter in adulthood. Int J Neuropsychopharmacol. (2019) 22:349–57. 10.1093/ijnp/pyz013 30911751 PMC6499245

[122] Diorio D Viau V Meaney MJ. The role of the medial prefrontal cortex (cingulate gyrus) in the regulation of hypothalamic-pituitary-adrenal responses to stress. J Neurosci. (1993) 13:3839–47. 10.1523/JNEUROSCI.13-09-03839.1993 8396170 PMC6576467

[123] Sullivan RM Gratton A. Lateralized effects of medial prefrontal cortex lesions on neuroendocrine and autonomic stress responses in rats. J Neurosci. (1999) 19:2834–40. 10.1523/JNEUROSCI.19-07-02834.1999 10087094 PMC6786056

[124] Fenster RJ Lebois LA Ressler KJ Suh J. Brain circuit dysfunction in post-traumatic stress disorder: from mouse to man. Nat Rev Neurosci. (2018) 19:535–51. 10.1038/s41583-018-0039-7 30054570 PMC6148363

[125] Hurley KM Herbert H Moga MM Saper CB. Efferent projections of the infralimbic cortex of the rat. J Comp Neurol. (1991) 308:249–76. 10.1002/cne.903080210 1716270

[126] Radley JJ Arias CM Sawchenko PE. Regional differentiation of the medial prefrontal cortex in regulating adaptive responses to acute emotional stress. J Neurosci. (2006) 26:12967–76. 10.1523/JNEUROSCI.4297-06.2006 17167086 PMC6674963

[127] Roddy DW Roman E Rooney S Andrews S Farrell C Doolin K. Awakening neuropsychiatric research into the stria medullaris: development of a diffusion-weighted imaging tractography protocol of this key limbic structure. Front Neuroanat. (2018) 12:e39. 10.3389/fnana.2018.00039 29867378 PMC5952041

[128] Roman E Weininger J Lim B Roman M Barry D Tierney P. Untangling the dorsal diencephalic conduction system: a review of structure and function of the stria medullaris, habenula and fasciculus retroflexus. Brain Struct Funct. (2020) 225:1437–58. 10.1007/s00429-020-02069-8 32367265

[129] Herman JP Ostrander MM Mueller NK Figueiredo H. Limbic system mechanisms of stress regulation: hypothalamo-pituitary-adrenocortical axis. Prog Neuropsychopharmacol Biol Psychiatry. (2005) 29:1201–13. 10.1016/j.pnpbp.2005.08.006 16271821

[130] Marrus N Belden A Nishino T Handler T Ratnanather JT Miller M. Ventromedial prefrontal cortex thinning in preschool-onset depression. J Affect Disord. (2015) 180:79–86. 10.1016/j.jad.2015.03.033 25881284 PMC4772729

[131] Zhang A Yang C Li G Wang Y Liu P Liu Z. Functional connectivity of the prefrontal cortex and amygdala is related to depression status in major depressive disorder. J Affect Disord. (2020) 274:897–902. 10.1016/j.jad.2020.05.053 32664030

[132] Kong L Chen K Tang Y Wu F Driesen N Womer F. Functional connectivity between the amygdala and prefrontal cortex in medication-naive individuals with major depressive disorder. J Psychiatry Neurosci. (2013) 38:417. 10.1503/jpn.120117 24148846 PMC3819156

[133] Wu F Tu Z Sun J Geng H Zhou Y Jiang X. Abnormal functional and structural connectivity of amygdala-prefrontal circuit in first-episode adolescent depression: a combined fMRI and DTI Study. Front Psychiatry. (2020) 10:e983. 10.3389/fpsyt.2019.00983 32116814 PMC7013238

[134] Tang Y Kong L Wu F Womer F Jiang W Cao Y. Decreased functional connectivity between the amygdala and the left ventral prefrontal cortex in treatment-naive patients with major depressive disorder: a resting-state functional magnetic resonance imaging study. Psychol Med. (2013) 43:1921–7. 10.1017/S0033291712002759 23194671

[135] Chai XJ Whitfield-Gabrieli S Shinn AK Gabrieli JD Castanón AN McCarthy JM. Abnormal medial prefrontal cortex resting-state connectivity in bipolar disorder and schizophrenia. Neuropsychopharmacology. (2011) 36:2009–17. 10.1038/npp.2011.88 21654735 PMC3158318

[136] Tang Y Ma Y Chen X Fan X Jiang X Zhou Y. Age-specific effects of structural and functional connectivity in prefrontal-amygdala circuitry in women with bipolar disorder. BMC Psychiatry. (2018) 18:1–8. 10.1186/s12888-018-1732-9 29871591 PMC5989351

[137] Mukherjee P Sabharwal A Kotov R Szekely A Parsey R Barch DM. Disconnection between amygdala and medial prefrontal cortex in psychotic disorders. Schizophr Bull. (2016) 42:1056–67. 10.1093/schbul/sbw012 26908926 PMC4903065

[138] MacMillan HL Georgiades K Duku EK Shea A Steiner M Niec A. Cortisol response to stress in female youths exposed to childhood maltreatment: results of the youth mood project. Biol Psychiatry. (2009) 66:62–8. 10.1016/j.biopsych.2008.12.014 19217075 PMC3816014

[139] Peckins MK Dockray S Eckenrode JL Heaton J Susman EJ. The longitudinal impact of exposure to violence on cortisol reactivity in adolescents. J Adolesc Health. (2012) 51:366–72. 10.1016/j.jadohealth.2012.01.005 22999837 PMC3457020

[140] Trickett PK Gordis E Peckins MK Susman EJ. Stress reactivity in maltreated and comparison male and female young adolescents. Child Maltreat. (2014) 19:27–37. 10.1177/1077559513520466 24482544

[141] Bevans K Cerbone A Overstreet S. Relations between recurrent trauma exposure and recent life stress and salivary cortisol among children. Dev Psychopathol. (2008) 20:257–72. 10.1017/S0954579408000126 18211737

[142] Bernard K Frost A Bennett CB Lindhiem O. Maltreatment and diurnal cortisol regulation: a meta-analysis. Psychoneuroendocrinology. (2017) 78:57–67. 10.1016/j.psyneuen.2017.01.005 28167370

[143] Kuhlman KR Vargas I Geiss EG Lopez? Duran NP. Age of trauma onset and HPA axis dysregulation among trauma-exposed youth. J Traum Stress. (2015) 28:572–9. 10.1002/jts.22054 26556544 PMC5403247

[144] Schwabe L Haddad L Schachinger H. HPA axis activation by a socially evaluated cold-pressor test. Psychoneuroendocrinology. (2008) 33:890–5. 10.1016/j.psyneuen.2008.03.001 18403130

[145] George ED Bordner KA Elwafi HM Simen AA. Maternal separation with early weaning: a novel mouse model of early life neglect. BMC Neurosci. (2010) 11:1–14. 10.1186/1471-2202-11-123 20920223 PMC2955691

[146] Portero-Tresserra M Gracia-Rubio I Cantacorps L Pozo OJ Gómez-Gómez A Pastor A. Maternal separation increases alcohol-drinking behaviour and reduces endocannabinoid levels in the mouse striatum and prefrontal cortex. Eur Neuropsychopharmacol. (2018) 28:499–512. 10.1016/j.euroneuro.2018.02.003 29478745

[147] Aisa B Tordera R Lasheras B Del Rio J Ramirez M. Effects of maternal separation on hypothalamic–pituitary–adrenal responses, cognition and vulnerability to stress in adult female rats. Neuroscience. (2008) 154:1218–26. 10.1016/j.neuroscience.2008.05.011 18554808

[148] Targum SD Nemeroff CB. The effect of early life stress on adult psychiatric disorders. Innov Clin Neurosci. (2019) 16:35. 31037228 PMC6450674

[149] Lippard ET Nemeroff CB. The devastating clinical consequences of child abuse and neglect: increased disease vulnerability and poor treatment response in mood disorders. Am J Psychiatry. (2020) 177:20–36. 10.1176/appi.ajp.2019.19010020 31537091 PMC6939135

[150] Rakofsky J Rapaport M. Mood disorders. Continuum. (2018) 24:804–27. 10.1212/CON.0000000000000604 29851879

[151] Carr A. Thematic review of family therapy journals 2012. J Fam Ther. (2013) 35:407–36. 10.1111/1467-6427.12021

[152] Nikkheslat N McLaughlin AP Hastings C Zajkowska Z Nettis MA Mariani N. Childhood trauma, HPA axis activity and antidepressant response in patients with depression. Brain Behav Immun. (2020) 87:229–37. 10.1016/j.bbi.2019.11.024 31794798 PMC7327513

[153] LeMoult J Ordaz SJ Kircanski K Singh MK Gotlib IH. Predicting first onset of depression in young girls: Interaction of diurnal cortisol and negative life events. J Abnorm Psychol. (2015) 124:850. 10.1037/abn0000087 26595472 PMC4662047

[154] Fischer S King S Papadopoulos A Hotopf M Young A Cleare A. Hair cortisol and childhood trauma predict psychological therapy response in depression and anxiety disorders. Acta Psychiatr Scand. (2018) 138:526–35. 10.1111/acps.12970 30302747

[155] Mayer SE Peckins M Kuhlman KR Rajaram N Lopez-Duran NL Young EA. The roles of comorbidity and trauma exposure and its timing in shaping HPA axis patterns in depression. Psychoneuroendocrinology. (2020) 120:104776. 10.1016/j.psyneuen.2020.104776 32593866 PMC7502500

[156] Lopez-Duran NL Kovacs M George CJ. Hypothalamic–pituitary–adrenal axis dysregulation in depressed children and adolescents: a meta-analysis. Psychoneuroendocrinology. (2009) 34:1272–83. 10.1016/j.psyneuen.2009.03.016 19406581 PMC2796553

[157] Knorr U Vinberg M Kessing LV Wetterslev J. Salivary cortisol in depressed patients versus control persons: a systematic review and meta-analysis. Psychoneuroendocrinology. (2010) 35:1275–86. 10.1016/j.psyneuen.2010.04.001 20447770

[158] Stetler C Miller GE. Depression and hypothalamic-pituitary-adrenal activation: a quantitative summary of four decades of research. Psychosom Med. (2011) 73:114–26. 10.1097/PSY.0b013e31820ad12b 21257974

[159] Murri MB Pariante C Mondelli V Masotti M Atti AR Mellacqua Z. HPA axis and aging in depression: systematic review and meta-analysis. Psychoneuroendocrinology. (2014) 41:46–62. 10.1016/j.psyneuen.2013.12.004 24495607

[160] Psarraki EE Kokka I Bacopoulou F Chrousos GP Artemiadis A Darviri C. Is there a relation between major depression and hair cortisol? A systematic review and meta-analysis. Psychoneuroendocrinology. (2020) 124:105098. 10.1016/j.psyneuen.2020.105098 33310696

[161] Fischer S Strawbridge R Vives AH Cleare AJ. Cortisol as a predictor of psychological therapy response in depressive disorders: systematic review and meta-analysis. Br J Psychiatry. (2017) 210:105–9. 10.1192/bjp.bp.115.180653 27908897

[162] Aas M Ueland T Inova A Melle I Andreassen OA Steen NE. Childhood trauma is nominally associated with elevated cortisol metabolism in severe mental disorder. Front Psychiatry. (2020) 11:391. 10.3389/fpsyt.2020.00391 32528319 PMC7247816

[163] Quidé Y Girshkin L Watkeys OJ Carr VJ Green MJ. The relationship between cortisol reactivity and emotional brain function is differently moderated by childhood trauma, in bipolar disorder, schizophrenia and healthy individuals. Eur Arch Psychiatry Clin Neurosci. (2020) 271:1089–109. 10.1007/s00406-020-01190-3 32926285

[164] Lu S Gao W Huang M Li L Xu Y. In search of the HPA axis activity in unipolar depression patients with childhood trauma: combined cortisol awakening response and dexamethasone suppression test. J Psychiatr Res. (2016) 78:24–30. 10.1016/j.jpsychires.2016.03.009 27049575

[165] Peng H Long Y Li J Guo Y Wu H Yang Y. Hypothalamic-pituitary-adrenal axis functioning and dysfunctional attitude in depressed patients with and without childhood neglect. BMC Psychiatry. (2014) 14:1–7. 10.1186/1471-244X-14-45 24548345 PMC3937002

[166] Cantave CY Langevin S Marin M-F Brendgen M Lupien S Ouellet-Morin I. Impact of maltreatment on depressive symptoms in young male adults: the mediating and moderating role of cortisol stress response and coping strategies. Psychoneuroendocrinology. (2019) 103:41–8. 10.1016/j.psyneuen.2018.12.235 30640036

[167] Suzuki A Poon L Papadopoulos AS Kumari V Cleare AJ. Long term effects of childhood trauma on cortisol stress reactivity in adulthood and relationship to the occurrence of depression. Psychoneuroendocrinology. (2014) 50:289–99. 10.1016/j.psyneuen.2014.09.007 25265282

[168] Burke HM Davis MC Otte C Mohr DC. Depression and cortisol responses to psychological stress: a meta-analysis. Psychoneuroendocrinology. (2005) 30:846–56. 10.1016/j.psyneuen.2005.02.010 15961250

[169] Ciufolini S Dazzan P Kempton MJ Pariante C Mondelli V. HPA axis response to social stress is attenuated in schizophrenia but normal in depression: evidence from a meta-analysis of existing studies. Neurosci Biobehav Rev. (2014) 47:359–68. 10.1016/j.neubiorev.2014.09.004 25246294

[170] Watson S Owen BM Gallagher P Hearn AJ Young AH Ferrier IN. Family history, early adversity and the hypothalamic-pituitary-adrenal (HPA) axis: mediation of the vulnerability to mood disorders. Neuropsychiatr Dis Treat. (2007) 3:647. Availalble online at: https://www.dovepress.com/family-history-early-adversity-and-thehypothalamic-pituitary-adrenal-peer-reviewed-fulltext-article-NDT 19300594 PMC2656301

[171] Mokhtari M Arfken C Boutros N. The DEX/CRH test for major depression: a potentially useful diagnostic test. Psychiatry Res. (2013) 208:131–9. 10.1016/j.psychres.2012.09.032 23291044

[172] Girshkin L Matheson SL Shepherd AM Green MJ. Morning cortisol levels in schizophrenia and bipolar disorder: a meta-analysis. Psychoneuroendocrinology. (2014) 49:187–206. 10.1016/j.psyneuen.2014.07.013 25108162

[173] Murri MB Prestia D Mondelli V Pariante C Patti S Olivieri B. The HPA axis in bipolar disorder: systematic review and meta-analysis. Psychoneuroendocrinology. (2016) 63:327–42. 10.1016/j.psyneuen.2015.10.014 26547798

[174] Aas M Pizzagalli DA Laskemoen JF Reponen EJ Ueland T Melle I. Elevated hair cortisol is associated with childhood maltreatment and cognitive impairment in schizophrenia and in bipolar disorders. Schizophr Res. (2019) 213:65–71. 10.1016/j.schres.2019.01.011 30660575

[175] Monteleone AM Cascino G Marciello F D'Agostino G Caivano V Monteleone P. Clinical and neuroendocrine correlates of childhood maltreatment history in adults with bipolar disorder. Bipolar Disord. (2020) 22:749–56. 10.1111/bdi.12923 32365252

[176] Mansueto G Faravelli C. Recent life events and psychosis: the role of childhood adversities. Psychiatry Res. (2017) 256:111–7. 10.1016/j.psychres.2017.06.042 28628791

[177] McCutcheon A Marques TR Howes OD. Schizophrenia—an overview. JAMA Psychiatry. (2020) 77:201–10. 10.1001/jamapsychiatry.2019.3360 31664453

[178] Carr CP Martins CMS Stingel AM Lemgruber VB Juruena MF. The role of early life stress in adult psychiatric disorders: a systematic review according to childhood trauma subtypes. J Nerv Ment Dis. (2013) 201:1007–20. 10.1097/NMD.0000000000000049 24284634

[179] Karanikas E Antoniadis D Garyfallos GD. The role of cortisol in first episode of psychosis: a systematic review. Curr Psychiatry Rep. (2014) 16:503. 10.1007/s11920-014-0503-7 25200986

[180] Berger M Kraeuter AK Romanik D Malouf P Amminger GP Sarnyai Z. Cortisol awakening response in patients with psychosis: systematic review and meta-analysis. Neurosci Biobehav Rev. (2016) 68:157–66. 10.1016/j.neubiorev.2016.05.027 27229759

[181] Zorn JV Schür RR Boks MP Kahn RS Joëls M Vinkers CH. Cortisol stress reactivity across psychiatric disorders: a systematic review and meta-analysis. Psychoneuroendocrinology. (2017) 77:25–36. 10.1016/j.psyneuen.2016.11.036 28012291

[182] Cullen AE Zunszain PA Dickson H Roberts RE Fisher HL Pariante CM. Cortisol awakening response and diurnal cortisol among children at elevated risk for schizophrenia: relationship to psychosocial stress and cognition. Psychoneuroendocrinology. (2014) 46:1–13. 10.1016/j.psyneuen.2014.03.010 24882153 PMC4065330

[183] Lange C Huber CG Fröhlich D Borgwardt S Lang UE Walter M. Modulation of HPA axis response to social stress in schizophrenia by childhood trauma. Psychoneuroendocrinology. (2017) 82:126–32. 10.1016/j.psyneuen.2017.03.027 28549268

[184] American Psychiatric Association. Bipolar and related disorders. In: Diagnostic and Statistical Manual of 1665 Mental Disorders (DSM-5). American Psychiatric Pub (2013).

[185] O'donovan MC Craddock N Norton N Williams H Peirce T Moskvina V. Identification of loci associated with schizophrenia by genome-wide association and follow-up. Nat Genet. (2008) 40:1053–55. 10.1038/ng.201 18677311

[186] Vazza G Bertolin C Scudellaro E Vettori A Boaretto F Rampinelli S. Genome-wide scan supports the existence of a susceptibility locus for schizophrenia and bipolar disorder on chromosome 15q26. Mol Psychiatry. (2007) 12:87–93. 10.1038/sj.mp.4001895 16969366

[187] Badner J Gershon E. Meta-analysis of whole-genome linkage scans of bipolar disorder and schizophrenia. Mol Psychiatry. (2002) 7:405–11. 10.1038/sj.mp.4001012 11986984

[188] Craddock N O'Donovan MC Owen MJ. Genes for schizophrenia and bipolar disorder? Implications for psychiatric nosology. Schizophr Bull. (2006) 32:9–16. 10.1093/schbul/sbj033 16319375 PMC2632175

[189] Levinson DF. The genetics of depression: a review. Biol Psychiatry. (2006) 60:84–92. 10.1016/j.biopsych.2005.08.024 16300747

[190] Laksono JP Sumirtanurdin R Dania H Ramadhani FN Perwitasari DA Abdulah R. Polymorphism of TPH2 gene rs120074175 is not associated with risk factors of schizophrenia. J Pharm Bioallied Sci. (2019) 11:S601. 10.4103/jpbs.JPBS_216_19 32148370 PMC7020838

[191] Doolin K Allers KA Pleiner S Liesener A Farrell C Tozzi L. Altered tryptophan catabolite concentrations in major depressive disorder and associated changes in hippocampal subfield volumes. Psychoneuroendocrinology. (2018) 95:8–17. 10.1016/j.psyneuen.2018.05.019 29787958

[192] Birur B Kraguljac NV Shelton RC Lahti AC. Brain structure, function, and neurochemistry in schizophrenia and bipolar disorder—a systematic review of the magnetic resonance neuroimaging literature. NPJ Schizophr. (2017) 3:15. 10.1038/s41537-017-0013-9 28560261 PMC5441538

